# Comparative Analysis and Phylogeny of the Complete Chloroplast Genomes of Nine *Cynanchum* (Apocynaceae) Species

**DOI:** 10.3390/genes15070884

**Published:** 2024-07-05

**Authors:** Erdong Zhang, Xueling Ma, Ting Guo, Yujie Wu, Lei Zhang

**Affiliations:** Key Laboratory of Ecological Protection of Agro-Pastoral Ecotones in the Yellow River Basin, National Ethnic Affairs Commission of the People’s Republic of China, College of Biological Science & Engineering, North Minzu University, Yinchuan 750021, China; zhangerdong0523@outlook.com (E.Z.); m17341237956@outlook.com (X.M.); gettyguo@foxmail.com (T.G.); plant-09324@hotmail.com (Y.W.)

**Keywords:** *Cynanchum*, chloroplast genomes, comparative analysis, phylogenomics

## Abstract

*Cynanchum* belongs to the Apocynaceae family and is a morphologically diverse genus that includes around 200 shrub or perennial herb species. Despite the utilization of CPGs, few molecular phylogenetic studies have endeavored to elucidate infrafamilial relationships within Cynanchum through extensive taxon sampling. In this research, we constructed a phylogeny and estimated divergence time based on the chloroplast genomes (CPGs) of nine *Cynanchum* species. We sequenced and annotated nine chloroplast (CP) genomes in this study. The comparative analysis of these genomes from these *Cynanchum* species revealed a typical quadripartite structure, with a total sequence length ranging from 158,283 to 161,241 base pairs (bp). The CP genome (CPG) was highly conserved and moderately differentiated. Through annotation, we identified a total of 129–132 genes. Analysis of the boundaries of inverted repeat (IR) regions showed consistent positioning: the rps19 gene was located in the IRb region, varying from 46 to 50 bp. IRb/SSC junctions were located between the trnN and ndhF genes. We did not detect major expansions or contractions in the IR region or rearrangements or insertions in the CPGs of the nine *Cynanchum* species. The results of SSR analysis revealed a variation in the number of SSRs, ranging from 112 to 150. In five types of SSRs, the largest number was mononucleotide repeats, and the smallest number was hexanucleotide repeats. The number of long repeats in the cp genomes of nine *Cynanchum* species was from 35 to 80. In nine species of *Cynanchum*, the GC3s values ranged from 26.80% to 27.00%, indicating a strong bias towards A/U-ending codons. Comparative analyses revealed four hotspot regions in the CPG, ndhA-ndhH, trnI-GAU-rrn16, psbI-trnS-GCU, and rps7-ndhB, which could potentially serve as molecular markers. In addition, phylogenetic tree construction based on the CPG indicated that the nine *Cynanchum* species formed a monophyletic group. Molecular dating suggested that *Cynanchum* diverged from its sister genus approximately 18.87 million years ago (Mya) and species diversification within the *Cynanchum* species primarily occurred during the recent Miocene epoch. The divergence time estimation presented in this study will facilitate future research on *Cynanchum*, aid in species differentiation, and facilitate diverse investigations into this economically and ecologically important genus.

## 1. Introduction

*Cynanchum* belongs to the Apocynaceae family and is a morphologically diverse genus that includes around 200 shrub or perennial herb species [1,2]. *Cynanchum* species are primarily found in eastern Africa; the Mediterranean region; and the tropical, subtropical, and temperate regions of Europe and Asia [3]. These plants can be utilized for medicinal purposes, food, as forage resources, and for managing wasteland and barren land [4,5]. Some species with climbing vines and tuberous roots have been traditionally used in Korean and Chinese medicine [4,5,6,7,8,9]. Various studies have shown that extracts from these plants can help prevent and treat conditions like rheumatoid arthritis, lumbago, abdominal pain, and traumatic injuries [8,10,11]. Previous research has mainly focused on the growth, development, medicinal properties, and breeding of *Cynanchum* [12,13,14,15]. Despite its economic and ecological importance, there is still much to learn about Cynanchum phylogeny, interspecific relationships, and evolutionary history [16,17]. While many studies have identified and examined phylogenetic relationships within the *Cynanchum* genus using DNA barcode fragments [18,19], there is a lack of research on chloroplast genome (CPG) phylogeny and lineage diversification.

Chloroplasts play a crucial role in photosynthesis and carbon fixation in plant cells. Advances in sequencing technologies have made whole-chloroplast genome sequencing more accessible [20,21,22]. The CPG is characterized by two large inverted repeat regions (IRa and IRb) separated by two single-copy regions, known as the large single-copy region (LSC) and the small single-copy region (SSC) [23]. Compared to DNA fragments, the plant CPG has a relatively small molecular size (107~218 kb) and moderate rates of nucleotide substitution, making it a valuable tool for species identification, phylogeny, and genetic diversity studies [24]. CPGs have recently been utilized for comparative and phylogenetic analyses, proving beneficial for species identification, genetic diversity assessment, nucleotide diversity evaluation, resolving phylogenetic relationships, and understanding evolutionary history. For instance, comparative analyses of CPGs have successfully revealed the phylogenetic relationships within Brassicaceae [25,26], Salicaceae [27], and the genus *Yulania* [28].

Despite the utilization of CPGs, few molecular phylogenetic studies have endeavored to elucidate infrafamilial relationships within *Cynanchum* through extensive taxon sampling. Previous studies mostly relied on single or limited molecular loci or faced constraints in sampling, resulting in an unclear understanding of the phylogenetic relationships and divergence timescales of the genus [29,30]. In this research, we conducted sequencing and alignment of the chloroplast genomes of nine *Cynanchum* species. Our primary goals were to (a) establish a phylogeny based on the CPGs of nine species from the Apocynaceae family; (b) estimate the divergence of the Cynanchum clade; and (c) investigate structural alterations in the PCGs of the sampled *Cynanchum* species.

## 2. Results

### 2.1. Genome Size and Features

**Figure 1:**
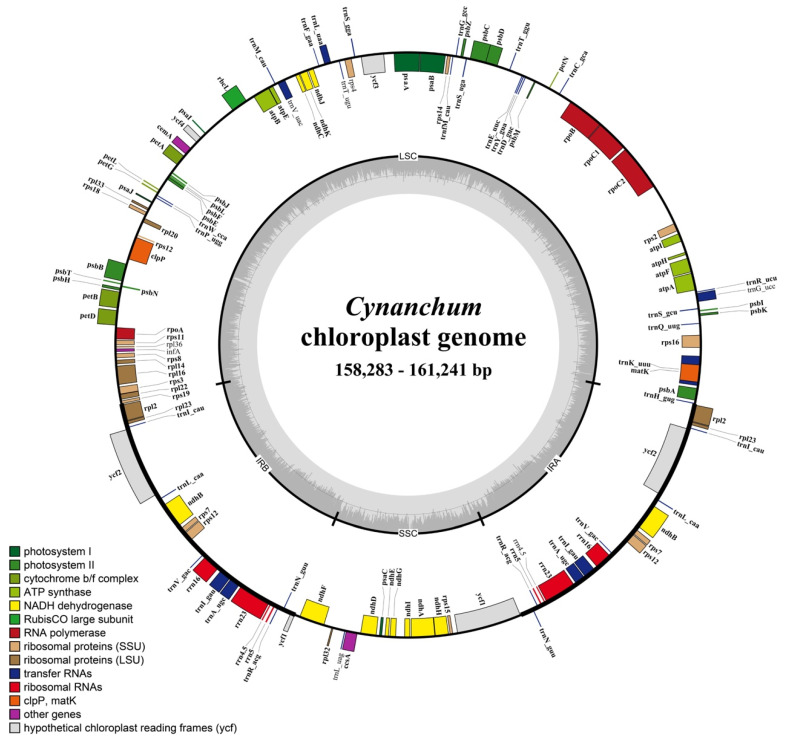
Gene map of the CPGs of nine *Cynanchum* species. Genes belonging to different functional groups are shown in different colors. The darker gray area in the inner circle indicates the GC content, and the lighter gray indicates the AT content of the genome. The thick lines indicate the extent of the inverted repeats (IRa and IRb) that separate the genomes into the small single-copy (SSC) and large single-copy (LSC) regions.

After quality control and pre-processing, a minimum of 6 Gb of whole-genome sequencing data were obtained for each of the nine species included in this study (Table 1). These clean reads were used to assemble complete chloroplast genomes using a reference-guided approach. All the newly assembled CP genomes displayed a typical quadripartite structure, with two IR regions separating the LSC and SSC regions (Figure 1).

For each of the nine *Cynanchum* species, the CP genome size ranged from 158,283 bp (*Cynanchum. acutum* subsp. *sibiricum*) to 161,241 bp (*C. wilfordii*) (Table 1). All the CPGs had a typical quadripartite circular structure (Figure 1) consisting of an LSC and an SSC region separated by a pair of IR regions (Figure 1 and Table 1). The LSC region’s length varied from 89,424 bp (*C. acutum* subsp. *sibiricum*) to 92,051 bp (*C. rostellatum*), and the lengths of the SSC and IR regions ranged from 18,281 bp (*C. thesioides*) to 20,943 bp (*C. wallichii*) and 23,834 bp (*C. rostellatum*) to 24,658 bp (*C. wilfordii*), respectively (Table 1). The GC content of the entire plasmid sequence and the LSC, SSC, and IR regions was similar across all *Cynanchum* CPGs. Specifically, the GC content of the entire CPGs sequence was 37.8–38.1%, while the GC content of the IR regions was 42.69–44.55%, which was higher than that of the LSC and SSC regions (35.53–36.24% and 30.2–32.57%, respectively; Figure S1 and Table S1). Additionally, the number of annotated genes in each CPG ranged from 129 (*C. acutum* subsp. *sibiricum*) to 133 (*C. wallichii*) and included 37 tRNA and 8 rRNA genes.

### 2.2. Comparative Genomics and Divergence Hotspots

**Figure 2:**
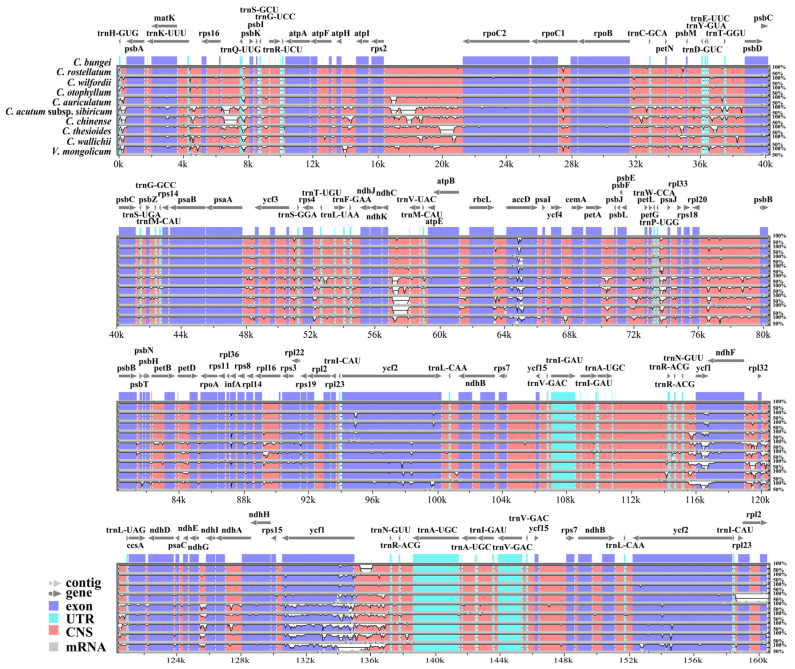
Sequence alignment of the CPGs of *Cynanchum* species. The alignment was performed using the mVISTA program and the *V. mongolicum* chloroplast genome was used as a reference. The Y-axis: the degree of identity ranging from 50 to 100%. Coding and non-coding regions were marked in blue and red, respectively. Black arrows indicated the position and direction of each gene. CNS: conserved non-coding sequences.

**Figure 3:**
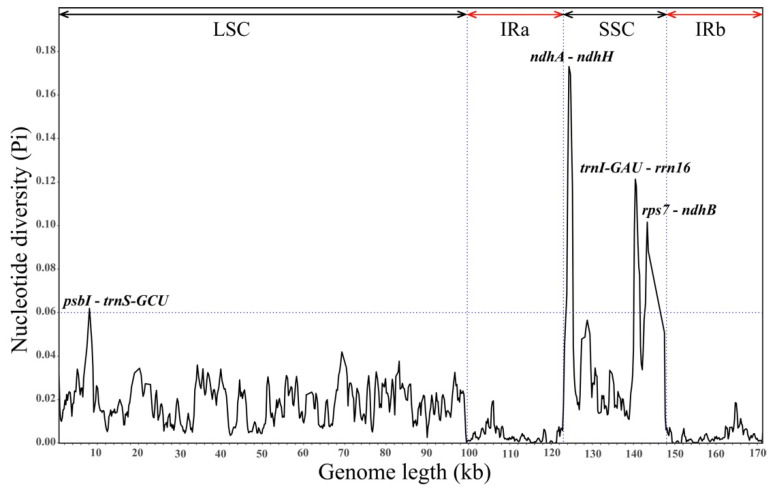
Sliding window test of nucleotide diversity (Pi) in the multiple alignments of nine *Cynanchum* species (window length: 600 bp; step size: 200 bp). X-axis: the position of the midpoint of the window; Y-axis: the nucleotide diversity of each window.

Using *V. mongolicum* as a reference, the CPGs of the nine *Cynanchum* species were visually compared with those obtained using the mVISTA online database. The results showed that the cp genomes of the nine *Cynanchum* species were conserved. Moreover, there were differences in the LSC and SSC regions compared with the IR region, and the same was true for the non-coding and coding regions (Figure 2). The regions with greater variation were located mainly in *rps*16-*trn*Q-UUG-*psb*K, *atp*H-*atp*I, *rps*2-*rpoC*2, *rpl*20-*psb*B, *rpl*16-*rps*3, *ndh*G-*ndh*I, and *ndh*F-*rpl*32 (Figure 2). The greatest variation among coding regions was observed in *acc*D, *ycf*2, and *ycf*1. No major genomic rearrangements or insertions were detected among the nine CPGs (Figure 2). DNA molecular markers are usually highly variable regions of sequences that can be used for the differentiation of relationships between species. Therefore, to further understand the DNA polymorphisms (Pi), mutation hotspot regions in the cp genomes of the nine *Cynanchum* plants were screened using DnaSP (Figure 3). Pi analysis revealed that the pi values ranged from 0 to 0.18, and the cp genome was relatively structurally conserved, small, and highly variable among the species. A total of six mutation hotspot regions (Pi > 0.06) were detected, and they can be used as potential molecular markers. Among these, *ndh*A-*ndh*H, *trn*I-GAU-*rrn*16, and *rps*7-*ndh*B were located in the SSC region; *psb*I-*trn*S-GCU was located in the LSC region (Figure 3). None of the mutation hotspots were located in the IR region.

### 2.3. Boundaries between IR and SC Regions

**Figure 4:**
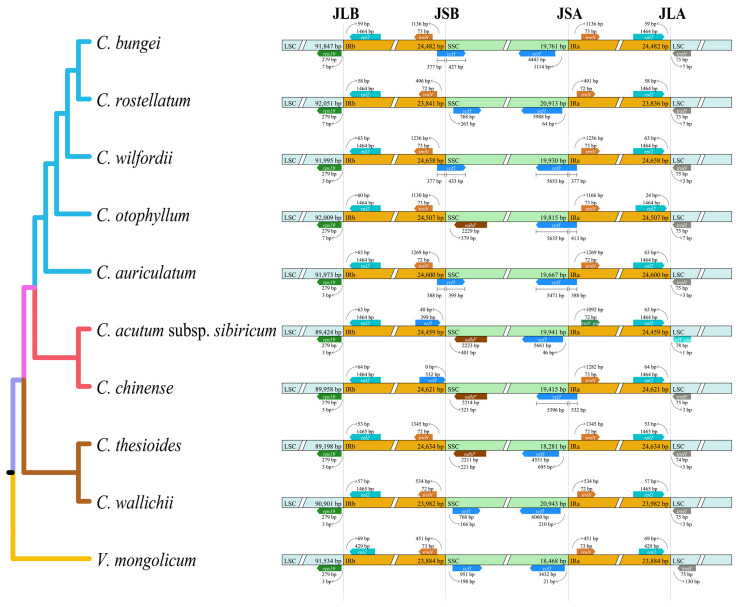
Comparisons of the borders of the large single-copy (LSC), small single-copy (SSC), and inverted repeat (IR) regions among the CPGs of nine *Cynanchum* species.

We analyzed the dynamics of the IR boundaries of the cp genomes of nine *Cynanchum* species. The results showed that *Cynanchum* chloroplast genomes were relatively conserved; however, some structural variations were still identified, especially at the boundaries between the IR and SSC regions (Figure 4), with the length of the rps19 gene located in the IRb region, varying from 46 to 50 bp. At both ends of the SSC region, IRb/SSC junctions were located between the trnN and ndhF genes. However, the ycf1 genes of *C. bungei*, *C. rostellatum*, *C. acutum* subsp. *sibiricum*, *C. thesioides,* and *C. wallichii* were located in the SSC region, and the ycf1 genes of the other four species were located in the LSC-IRb boundary. SSC/IRa was found in the ycf1 gene, with the length of the ycf1 gene located in the IRa region varying from 377 to 532 bp. Here, trnH was shown to be the first gene in the LSC region at the junction between IRa and LSC (i.e., IRa/LSC). At the other end of the LSC region, the IRb/LSC junctions were located between the rps19 and rpl2 genes in all Cynanchum species.

### 2.4. Repeat Identification

**Figure 5:**
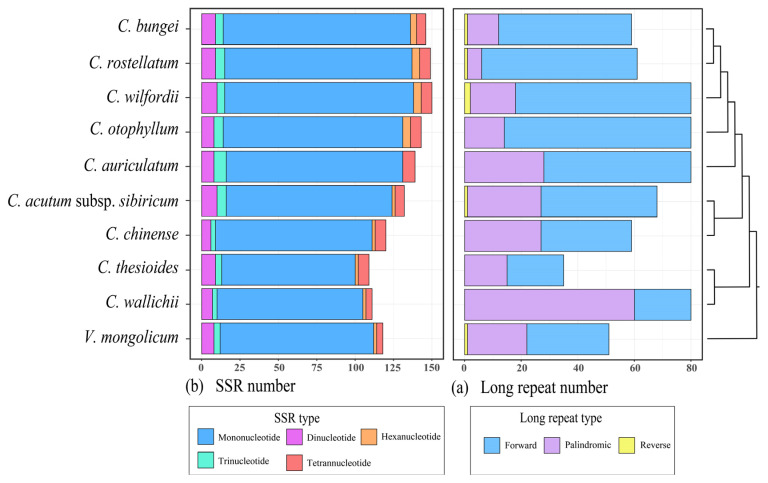
Repeat analysis of chloroplast genomes of *Cynanchum* species. (**a**) SSR statistics of *Cynanchum* species. (**b**) Long repeat statistics of *Cynanchum* species. Different types of repeats are indicated by different colors.

**Figure 6:**
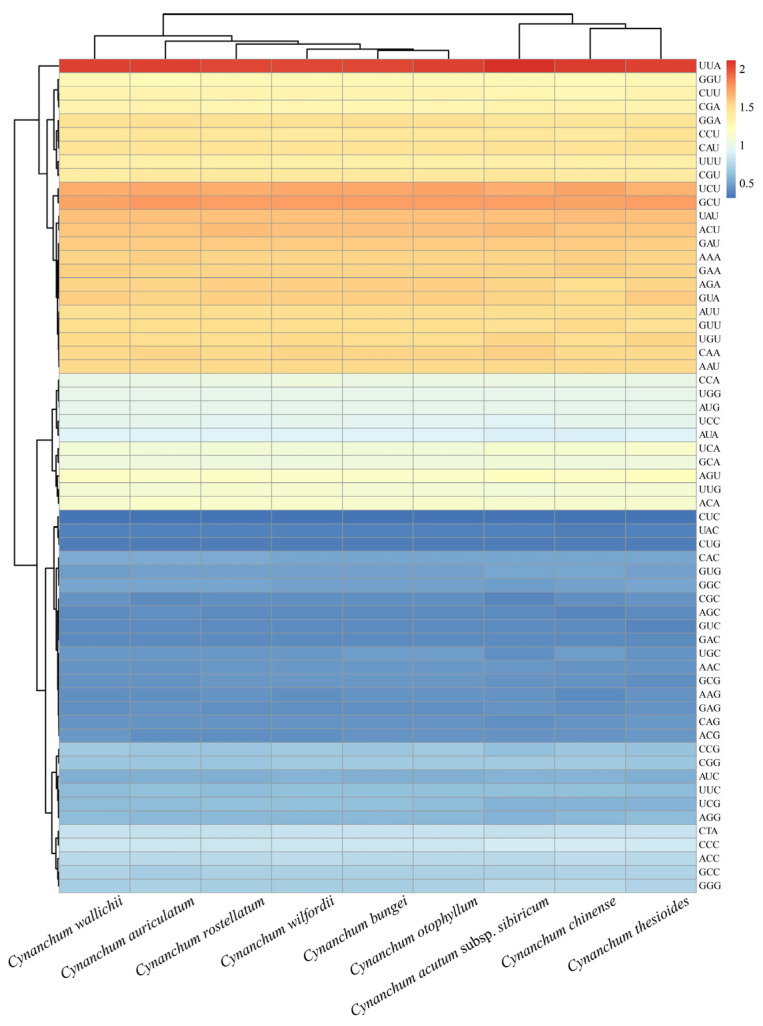
Heatmap of the RSCU values among nine *Cynanchum* species.

The MISA v. 1.0 software was utilized to detect simple sequence repeats (SSRs) in nine cp genomes of *Cynanchum* (Figure 5a; Table S1). The results of SSR analysis revealed a variation in the number of SSRs, ranging from 112 to 150. These SSRs were predominantly located in the LSC and SSC regions of the gene spacer among the five types of SSRs, and the largest number was mononucleotide repeats, accounting for 82.3%, followed by dinucleotide and tetranucleotide repeats, accounting for 6.3% and 4.9%, respectively. The smallest number was hexanucleotide repeats, accounting for only 2.6%. We examined the number and distribution of long repeats in the cp genomes of nine *Cynanchum* species, which ranged from 35 to 80, with an average of 67 repeats, mainly in the IR and LSC regions (Figure 6; Table S2). Five *Cynanchum* species contained only forward and palindromic repeats, namely *C. otophyllum*, *C. auriculatum*, *C. chinense*, *C. thesioides,* and *C. wallichii*.

### 2.5. Codon Usage Analysis

The amino acid frequency, the number and bias of codon usage, and RSCU were investigated among the nine *Cynanchum* species cp genomes. The results indicated that 21 different amino acids were encoded in the cp genome, and a comprehensive set of 64 codons were deduced. Among these codons, 32 were frequently utilized in various *Cynanchum* species (Tables S3–S11). A total of 30 codons displayed RSCU values > 1, 29 of which had A or U terminal nucleotides. The rest of the 34 codons had RSCU values ≤ 1, where 31 of these ended in G or C nucleotides. Additionally, the codon UUA had the highest frequency, followed by GCU, while CUC was the least common (Figure 6). Leucine and aspartic acid had the highest and lowest number of codons, respectively. Moreover, unlike other amino acids, which were encoded by at least two synonymous codons, methionine and tryptophan were encoded by only one respective codon. The GC content of synonymous third codon positions (GC3s) is correlated with codon bias to evaluate codon usage patterns. In nine species of *Cynanchum*, the GC3s values ranged from 26.80% to 27.00%, indicating a strong bias towards A/U-ending codons. In addition, both the frequency of optical codons and the codon adaptation index were less than 0.4, and the effective proportion of codons ranged from 49.80% to 50.05% (Table S12). The codon usage of cp genomes in *C. wallichii*, *C. auriculatum*, and *C. thesioides* was relatively similar (Figure 6), suggesting that a minor bias existed in codon use across the nine *Cynanchum* species.

### 2.6. Phylogenetic Analyses and Divergence Time Estimation

**Figure 7:**
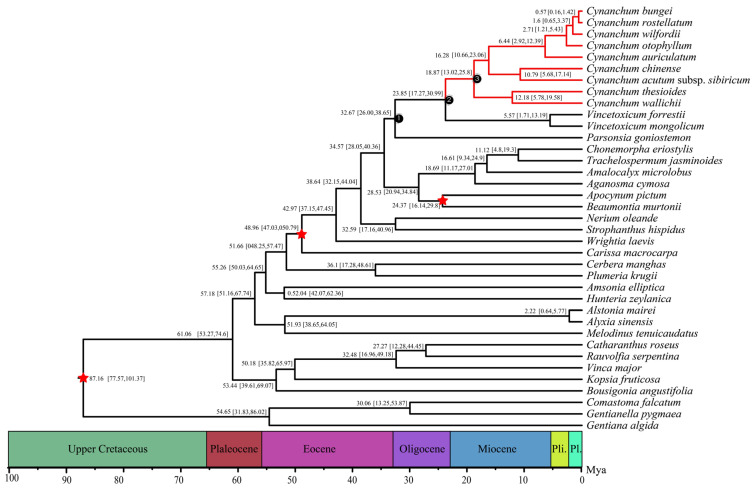
Phylogeny and clade divergence of Apocynaceae and outgroups based on 60 PCG protein-coding genes. Stars indicate time constrations in this analysis in this analysis. Geological periods are marked with background colors. Mya: million years ago; Pal: Paleocene; Pli: Pliocene; Pl: Pleistocene.

To infer the phylogenetic relationships of the 36 Apocynaceae species, we included 3 Gentianaceae species whose PCGs are publicly available in the GenBank database. These species (*Comastoma falcatum*, *Gentianella pygmaea,* and *Gentiana algida*) were used as the outgroup for phylogenetic analyses. The final concatenated dataset included 60 plastid genes and 43,362 sites, after trimming poorly aligned regions and gaps with missing genes (Table S13). In the phylogenetic trees, maximum likelihood (ML) analyses supported similar relationships to those indicated by maximum parsimony and Bayesian analyses (BI). The ML and BI analyses of each dataset contained nine internal clades with high bootstrap support (BS = 100%) and posterior probability (PP = 1) values (Figure 7 and Figures S2–S5). There were nine species of *Cynanchum* and two species of *Vincetoxicum* and *Parsonsia goniostemon* gathered into one clade, in which *Parsonsia goniostemon* resolved (BS; PP = 100%, 1) as a sister to *Cynanchum* plus *Vincetoxicum*. Further, in the CDS phylogeny, *Cynanchum* species were clustered on one large branch, confirming that the independence of this genus is highly supported (BS; PP = 100%, 1) (Figure 7 and Figures S2–S5).

We estimated the divergence timescales of the *Cynanchum* according to the time constrations of the gene tree constructed based on 60 plastid genes. The split between *Cynanchum* and its sister group was estimated to have occurred 23.85 Mya. The crown ages of all subclades in the genus *Cynanchum* were dated mainly within the Miocene, suggesting that numerous species of this genus originally diversified in the recent past (18.87 Mya) (Figure 7).

## 3. Discussion

Recent studies have reported that the CPG of seed plants range in size from 107 kb in Pinaceae to 170 kb in Geraniaceae, with the IR region typically spanning 20–30 kb [31,32]. In the present study, a comparative analysis of the CPGs indicated that those of nine *Cynanchum* species were ranging from 158,283 bp (*C. acutum* subsp. *sibiricum*) to 161,241 bp (*C. wilfordii*), at the larger end of the spectrum for seed plant organelle genomes. All *Cynanchum* species comprising the LSC region (89,424–92,05 bp), SSC region (18,281–20,943 bp), and two identical IR regions (23,834–24,658 bp) were similar to other vascular plants. The GC content of the entire plastid sequence and the LSC, SSC, and IR regions was similar across all *Cynanchum* CPGs. In agreement with numerous studies on angiosperms, the IR regions exhibited the highest GC content [26]. The conversion between sequences and higher GC content may contribute to the greater conservation of IR regions [27,28]. In angiosperms, the IR region is relatively conserved in sequence and structure. The narrowing and widening of its edges are not only important factors for length variation, but also the main cause of the emergence of pseudogenes [33,34]. While cp genes have evolved slowly and are relatively conserved in terms of sequence and structure, boundary contraction and expansion in the IR regions are common phenomena. In our study, we analyzed nine CPGs within the highly conserved *Cynanchum* species and noticed that no major expansions or contractions occurred in the IR regions.

Highly variable regions offer valuable phylogenetic information. For example, variable regions aid in species kinship identification and gene pool construction [25,26,35,36,37]. A good DNA barcode must be a short, representative DNA fragment with high variability and amenability to amplification [38]. In this study, both the sequence and structure of *Cynanchum* CPGs were highly conserved. mVISTA analysis revealed that most of the variation in nucleotide sequences occurred in non-coding regions, consistent with previous reports, suggesting this variation as a common feature of angiosperms [39,40,41]. In the *Cynanchum*, several highly variable regions, such as accD, ycf2, and ycf1, are recognized as potential DNA barcoding sites [42,43]. A comparable number of SSRs and long repeats were identified in nine *Cynanchum* species. However, the types of SSRs and long repeats differed among the species. These repeats were predominantly found in the intergenic spacer (IGS) of the large single-copy (LSC) region. Mononucleotide repeats were the most common types of SSRs, while forward and palindromic repeats were the predominant types of long repeats (Figure 5; Tables S1 and S2). In addition, our nucleotide diversity (Pi) analysis led to the identification of five highly variable regions with Pi values greater than 0.06, including three SSC regions (ndhA-ndhH, trnI-GAU-rrn16 and rps7-ndhB) and one LSC region (psbI-trnS-GCU). None of the mutation hotspots were located in the IR region. In conclusion, these mutation hotspot regions play an important role in the identification and characterization of *Cynanchum* plant species.

In nature, plants are often influenced by environment. Some genes may be subject to positive selection in response to environmental changes. In protein synthesis, codons represent the rule of a certain amino acid [44]. In this study, we found over 90% (RSCU ≥ 1) of *Cynanchum* codons terminated in A/U, and the GC3s value ranged from 26.80% to 27.00%. *Cynanchum* exhibited a high coding efficiency and a strong preference for A/U termination codons, possibly owing to the overall high AT content in the cp genome. This trend was also evident in other angiosperms [45]. Our investigation of *Cynanchum* genomes revealed that non-coding regions displayed more substantial variation than coding regions. Four highly variable regions in non-coding regions identified in this study effectively distinguished the most common *Cynanchum* species. These findings aligned with earlier studies of *Cynanchum* [36]. Non-coding regions evolve rapidly and contain valuable variations for genus phylogenetic analysis [37]. Therefore, the significance of non-coding regions in the cp genome for identifying *Cynanchum* species should be underscored.

The CPGs are central to molecular biology research and have become a prominent focus. In particular, species identification, phylogenetic relationships, and the reconstruction of evolutionary history via whole-genome sequencing have become important tools because of improvements in sequencing technology and low costs. Phylogenetic trees constructed based on a single or a few gene sequences often yield inconsistent or even conflicting topologies due to variations in evolutionary rates and horizontal shifts between genes. This complication affects the determination of accurate evolutionary relationships among species [46,47]. In this study, we constructed a phylogenetic tree using the BI and ML methods. The CPGs of nine *Cynanchum* species converged into branches with high support (Figures S4 and S5). Notably, *Parsonsia goniostemon* resolved (BS; PP = 100%, 1) as a sister to *Cynanchum* plus *Vincetoxicum*. The phylogenetic result was consistent with previous findings in Apocynaceae species [17,18,19,30], indicating that high-resolution CPG sequences offer valuable resources for extensive research on the genetic phylogeny and species identification of *Cynanchum* spp. Furthermore, further studies on *Cynanchum* are warranted, particularly to validate the relationships of this genus with other sister genera, such as *Vincetoxicum*. Expanding the number of cp genomes of *Cynanchum* and Apocynaceae will yield deeper insights into the evolution of this ecologically and economically important phytogroup.

To calibrate the divergence and origin of *Cynanchum*, we used 60 highly conserved and stable alignment plastid genes (Figure 7). We also used fossil date and time constrations to estimate the diversification. While the estimated ages should be used with caution, our findings indicate that the *Cynanchum* and Vincetoxicum diverged from the sister genus around 32.67 Mya, and the two *Cynanchum* and Vincetoxicum diverged 23.85 and 18.87 Mya, suggesting relatively late clade diversifications. Specifically, most species diversification within the subclades of *Cynanchum*, as estimated from these plastid genes, appeared to have occurred in the recent past, mostly after 20 Mya. The divergence timescales estimated here for the major subclades will serve as a basic timescale for diverse studies on this economically and ecologically important genus.

## 4. Conclusions

We analyzed the complete CPGs of nine *Cynanchum* species and found that all exhibited a quadripartite structure, typical of most angiosperms. The CPG arrangement was highly conserved, with great sequence variation observed in the SSC region compared with that in the IR region. We did not detect major expansions or contractions in the IR region, nor did we find any rearrangements or insertions in the CPGs of the nine *Cynanchum* species. We identified highly variable regions within *Cynanchum* that are likely to be useful for species delimitation. Mononucleotide repeats were the most common types of SSRs, while forward and palindromic repeats were the predominant types of long repeats. *Cynanchum* exhibited a high coding efficiency and a strong preference for A/U termination codons. Phylogenetic tree construction based on the CPGs showed that all nine *Cynanchum* species formed a monophyletic group, divided into two typical subbranches and three minor branches. Molecular dating suggested that *Cynanchum* diverged from its sister genus around 23.85 Mya, with species diversification of the *Cynanchum* species of China occurring mainly within the recent Pliocene epoch. Overall, our findings and the estimated divergence times will facilitate future studies on *Cynanchum*, assist in species differentiation, and facilitate diverse studies of this economically and ecologically significant genus.

## 5. Materials and Methods

### 5.1. Taxon Sampling, DNA Extraction, and PCG Sequencing

A total of 37 PCGs representing Apocynaceae and related families were included in this study (Table S14). Thirty-four PCGs from Apocynaceae were selected, including nine *Cynanchum* species found in China. Further, three additional PCGs from related families in Gentianaceae were chosen as outgroups for phylogenetic analysis. Among these 37 PCGs, 3 complete PCGs were newly sequenced, and the others were obtained from GenBank (Table S14). The leaves used in this study were collected from natural populations in China. The plant materials and specimens were deposited in the Herbarium of North Minzu University (NMU; Yinchuan, China). For each species, we extracted total DNA from dried leaves and preserved them in silica gel using the CTAB protocol [48]. Paired-end libraries with an insert size of 500 base pairs (bp) were constructed by Illumina (Qingdao, China) following sequencing with a HiSeq × Ten System (Jizhi, Qingdao, China).

### 5.2. Chloroplast Genome and Annotation

At least two gigabases (Gb) of 2 × 150 bp short read data were generated for each sample. Reads with quality scores of less than 7 and with more than 10% ambiguous nucleotides were filtered. The remaining reads were assembled using NOVOPlasty version 2.7.2 [49] software. The contigs were aligned into sequence in Geneious version 9.1.8 [50] software using the *V. mongolicum* PCG as a reference. The PCGs were annotated using Plann version 1.1 [51]. Protein-coding genes were extracted using customized Python scripts. The alignment of chloroplast genes across all species was performed using PRANK version 130410 [52] software. Poorly aligned regions were trimmed using Gblocks version 0.91b [53] with the option “−t = c,” selected to set the type of sequence to codons. Genes that were absent in at least one species were excluded, and the aligned sequences were combined into a super matrix. Additionally, circular maps of the CP genomes were created using OGDRAW version 1.2 [54], and all annotated CP genomes were submitted to GenBank [55].

### 5.3. Comparative Genomics and Structural Analyses

The structural variation and identification of arrangement events across *Cynanchum* was conducted for the nine CPGs of *Cynanchum*. The results of the comparative analysis of the CPGs were visualized with the mVISTA program [56], and the annotated CPG of *V. mongolicum* was used as the reference in the LAGAN mode [57]. The junction sites of the four structural regions (IRA, LSC, SSC, and IRB) and adjacent genes in nine *Cynanchum* PCGs were visualized and compared using IRscope v0.1.R [58] software to obtain a macroscopic view of the CP genome structure. Following sequence alignment, nucleotide diversity (Pi) analysis of the CP genome was performed using DnaSP version 6.0 [59].

### 5.4. Repeat Sequence and Codon Preference Analyses

The Geneious 9.1.8 software [50] was employed to conduct a GC content analysis. Furthermore, the REPuter program (https://bibiserv.cebitec.uni-bielefeld.de/reputer accessed on 6 July 2016) was utilized to recognize dispersed repeat sequences, including forward (F), complementary (C), palindromic (P), and reverse (R) [60], with the setting of >30 bp, ≥90% sequence identity, and a Hamming distance at 3. Simple sequence repeats (SSRs) in the cp genomes were analyzed on the MISA-web (http://pgrc.ipk-gatersleben.de/misa/ accessed on 5 August 2016) [61], and those with different repeat units were regarded as hexanucleotides, pentanucleotides, tetranucleotides, trinucleotides, dinucleotides, or mononucleotides. Additionally, the amino acid usage frequency and relative synonymous codon usage (RSCU) were identified via the CodonW 1.4.2 software [62]. Lastly, TBtools, a software that integrates various biological data handling tools [63], generated a heatmap of the RSCU values.

### 5.5. Phylogenetic Inference and Divergence Time Estimation

We generated two datasets for phylogenetic analysis: a protein-coding region (CDS) set and a whole PCG (WP) set. Protein-coding genes (PCGs) were extracted from the GenBank formatted file containing 37 PCGs using customized Perl scripts that removed the start and end codons. After excluding possible pseudogenes, 60 PCGs were retained in all species. Each PCG was aligned using PRANK version130410 based on the translated amino acid sequences. Genes that had been lost in at least one species were discarded, and then, the remaining aligned sequences were concatenated into a super matrix. Independent phylogenetic analyses were performed for each dataset (CDS and WP) using the maximum likelihood (ML) and Bayesian inference (BI) methodologies. We used RAxML version 8.1.24 [64] to conduct ML analyses with a general time reversible model with a gamma distribution (GTR + Γ). The best-scoring ML tree was obtained using the rapid hill-climbing algorithm (i.e., the option “-f d”) with 1000 bootstrap replicates. The optimal model (GTR + I + G) was identified using jModeltest 2.1.10 software, and BI analysis was conducted using MrBayes version 3.2.6 [65]. Additionally, FigTree version 1.4.2 [66] was used to visualize phylogeny.

We estimated divergence times from the PCG dataset using an approximate likelihood method, as implemented in MCMCtree in PAML version 4 [67] software, with independent relaxed-clock and birth–death sampling [68] strategies. Fossil dates were used as calibration points to reduce bias for more accurate age estimates [69]. In order to estimation the divergence time of *Cynanchum*, we used one fossil and two other time constrations to calibrate the phylogeny: (1) the root of the phylogeny occurred during the upper Cretaceous (~89 Mya, 95% HPD = 78–102 Mya) [70]. According to the ancient seed fossil of *Carissa manghas* [70], 47.02–50.78 Mya was the age range assigned to the split between *Carissa manghas* and its sister group [71]. (3) The split between *Apocynum* and *Beaumontia* was assigned an age range of 10.29–29.59 Mya as previously estimated [72]. The best-fit GTR + Γ model was selected, and the prior of the substitution rate (rgene) was modeled by a Γ distribution as Γ (2, 200, 1). We set parameters for the birth–death process with species sampling and σ^2^ values of (1, 1, 0.1) and G (1, 10, 1), respectively. We executed the MCMC runs for 2000 generations as burn-in and then sampled every 750 generations until 20,000 samples were obtained. We compared two MCMC runs for convergence using random seeds and obtained similar results.

## Figures and Tables

**Table 1:** General information and comparison of chloroplast genomes of *Cynanchum* species.

Taxa	*C. auriculatum*	*C. bungei*	*C. chinense*	*C. otophyllum*	*C. rostellatum*	*C. acutum* subsp. *sibiricum*	*C*. thesioides	*C. wallichii*	*C. wilfordii*
Total cpDNA size (bp)	160,840 bp	160,572 bp	158,615 bp	160,874 bp	160,641 bp	158,283 bp	156,747	159,808 bp	161,241 bp
LSC size (bp)	91,737	91,847	89,958	92,009	92,051	89,424	89,198	90,901	91,995
SSC size (bp)	19,667	19,761	19,415	19,815	20,922	19,713	18,281	20,943	19,930
IR size (bp)	24,600	24,482	24,621	24,507	23,834	24,573	24,634	23,982	24,658
Number of genes	132	131	132	131	132	129	132	133	132
Protein-coding genes	87	86	87	86	87	84	87	88	87
rRNA genes	8	8	8	8	8	8	8	8	8
tRNA genes	37	37	37	37	37	37	37	37	37
LSC GC%	35.57	36.1	35.53	35.64	36.08	36.15	36.24	36.15	35.62
SSC GC%	31.46	30.2	31.88	31.56	32.11	32.27	32.57	31.92	31.31
IR GC%	42.75	44.55	42.77	42.69	43.68	43.23	43.39	43.69	42.75
GC content(%)	37.8	37.8	37.8	37.8	37.8	37.9	38.1	37.9	37.8
Accession Number	KT220734	OK271106	MW415427	OQ587923	OL689165	OQ390041	MW864598	OQ198623	KT220733

## Data Availability

The original data presented in the study are openly available in https://www.ncbi.nlm.nih.gov, and accession numbers are listed in Table S14.

## Supplementary Materials

The following supporting information can be downloaded at https://www.mdpi.com/article/10.3390/genes15070884/s1: Table S1. Statistics of SSR analysis results from nine *Cynanchum* species. Table S2. Statistics of long repeats from nine Cynanchum species. Table S3. Codons in cp genome of *Cynanchum auriculatum.* Table S4. Codons in cp genome of *Cynanchum bungei.* Table S5. Codons in cp genome of *Cynanchum chinense.* Table S6. Codons in cp genome of *Cynanchum otophyllum.* Table S7. Codons in cp genome of *Cynanchum rostellatum.* Table S8. Codons in cp genome of *Cynanchum acutum* subsp. *sibiricum.* Table S9. Codons in cp genome of *Cynanchum thesioides.* Table S10. Codons in cp genome of *Cynanchum wallichii.* Table S11. Codons in cp genome of *Cynanchum wilfordi*i. Table S12. GC content at different positions of CDS sequence codon. Table S13. Comparison of plastid protein-coding genes. Table S14. Species and data description of plastomes used in this study. Figure S1. Changes in plastid GC content of nine *Cynanchum* species. This graph shows the total GC content (red bar) and the third codon position GC content (green bar) of each species. Figure S2. Phylogenetic tree obtained using the maximum likelihood (ML) methods for the *Cynanchum* species based on 60 PCGs. Figure S3. Phylogenetic tree obtained using the Bayesian inference (BI) methods for the ***C****ynanchum* species based on 60 PCGs. Figure S4. Phylogenetic tree obtained using the maximum likelihood (ML) methods for the *Cynanchum* species based on complete CPGs. Figure S5. Phylogenetic tree obtained using the Bayesian inference (BI) methods for the *Cynanchum* species based on complete CPGs.
