# Supplementary figures and images for: Comparative Analysis and Phylogeny of the Complete Chloroplast Genomes of Nine Cynanchum (Apocynaceae) Species
Source: Genes (Basel). 2024 Jul 5;15(7):884. doi: 10.3390/genes15070884 (PMC11275380; doi:10.3390/genes15070884)

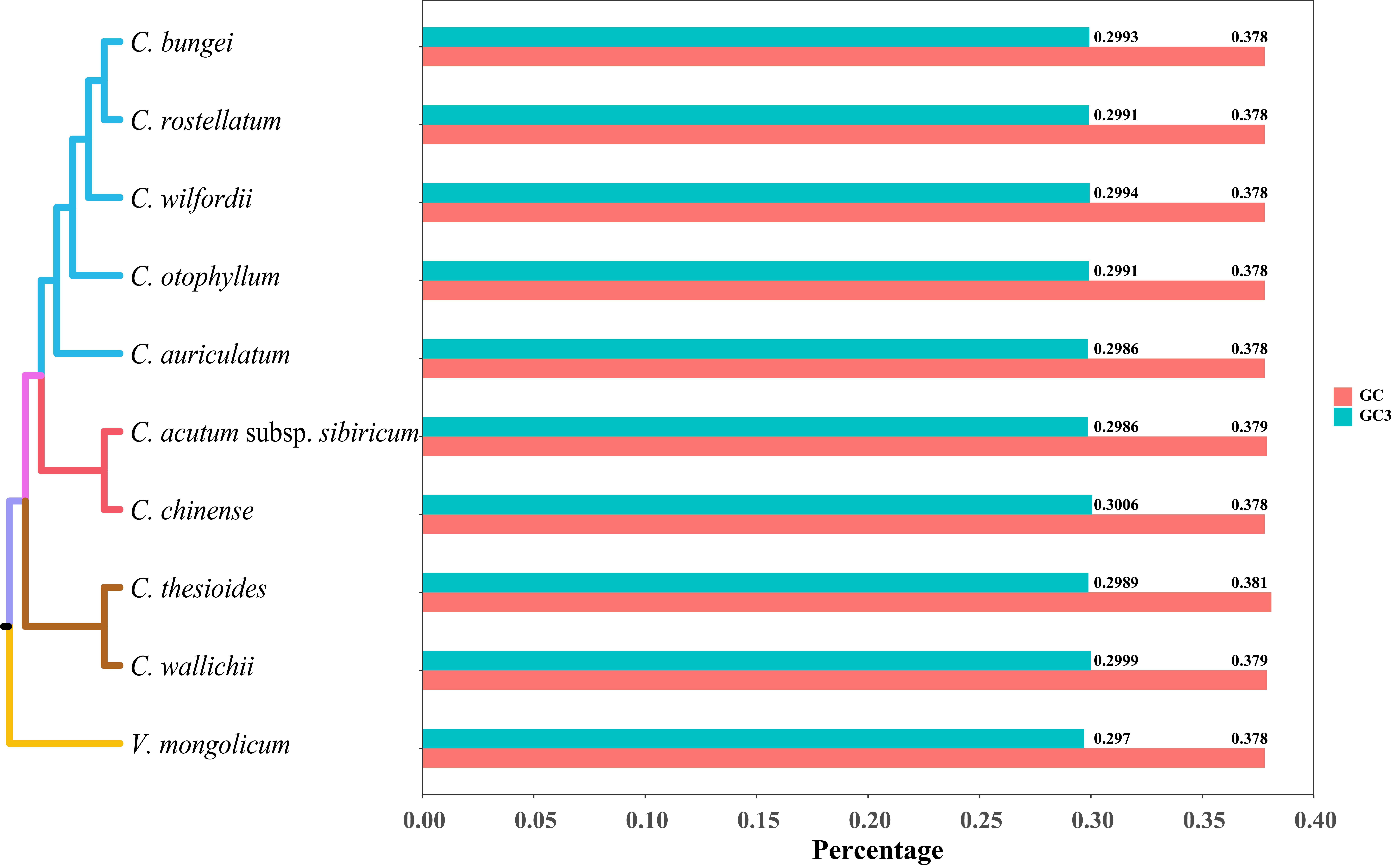

Supplement: Supplementary file 1 [file genes-15-00884-s001.zip › Figure S1.jpg]

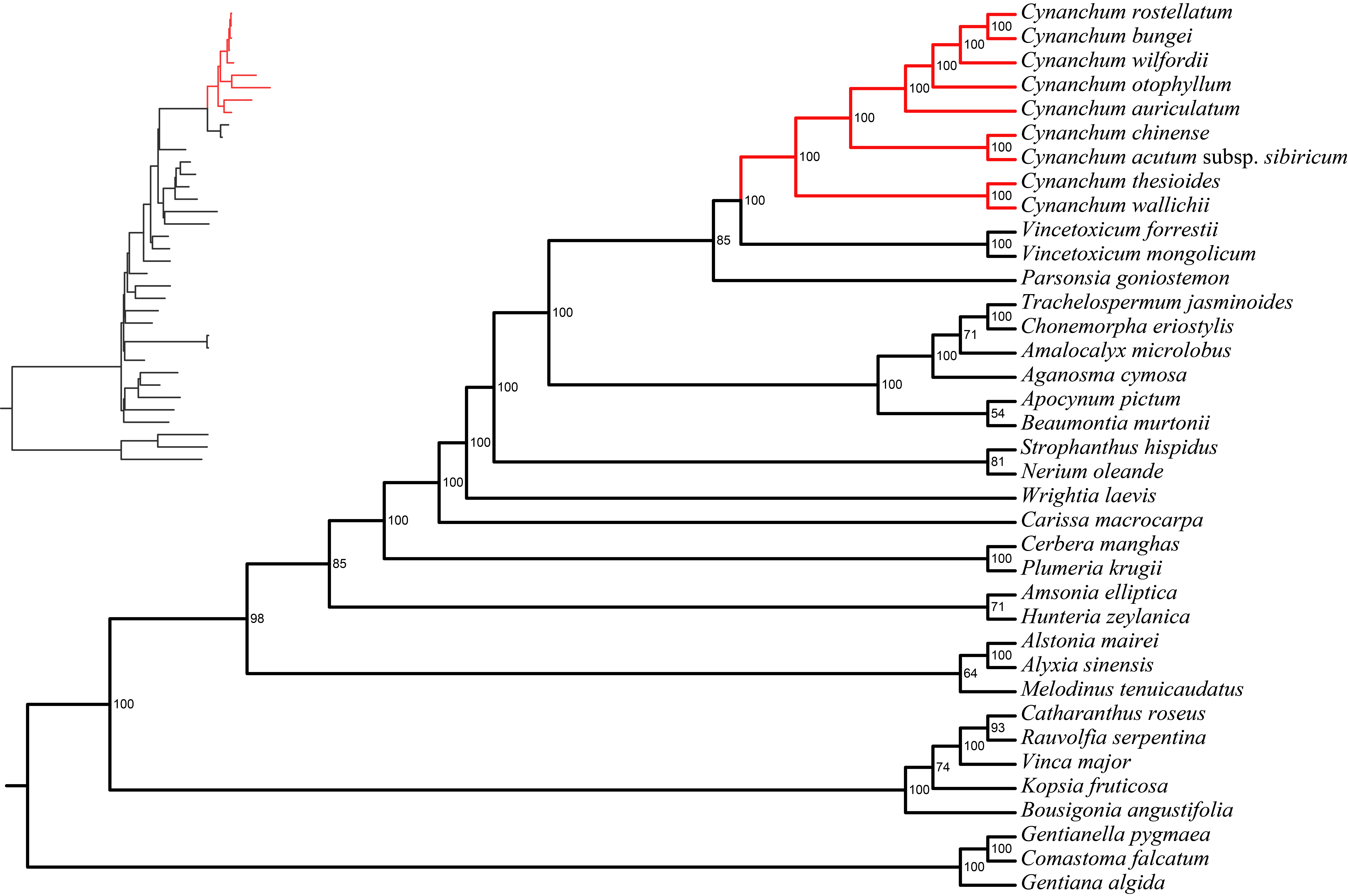

Supplement: Supplementary file 1 [file genes-15-00884-s001.zip › Figure S4.jpg]

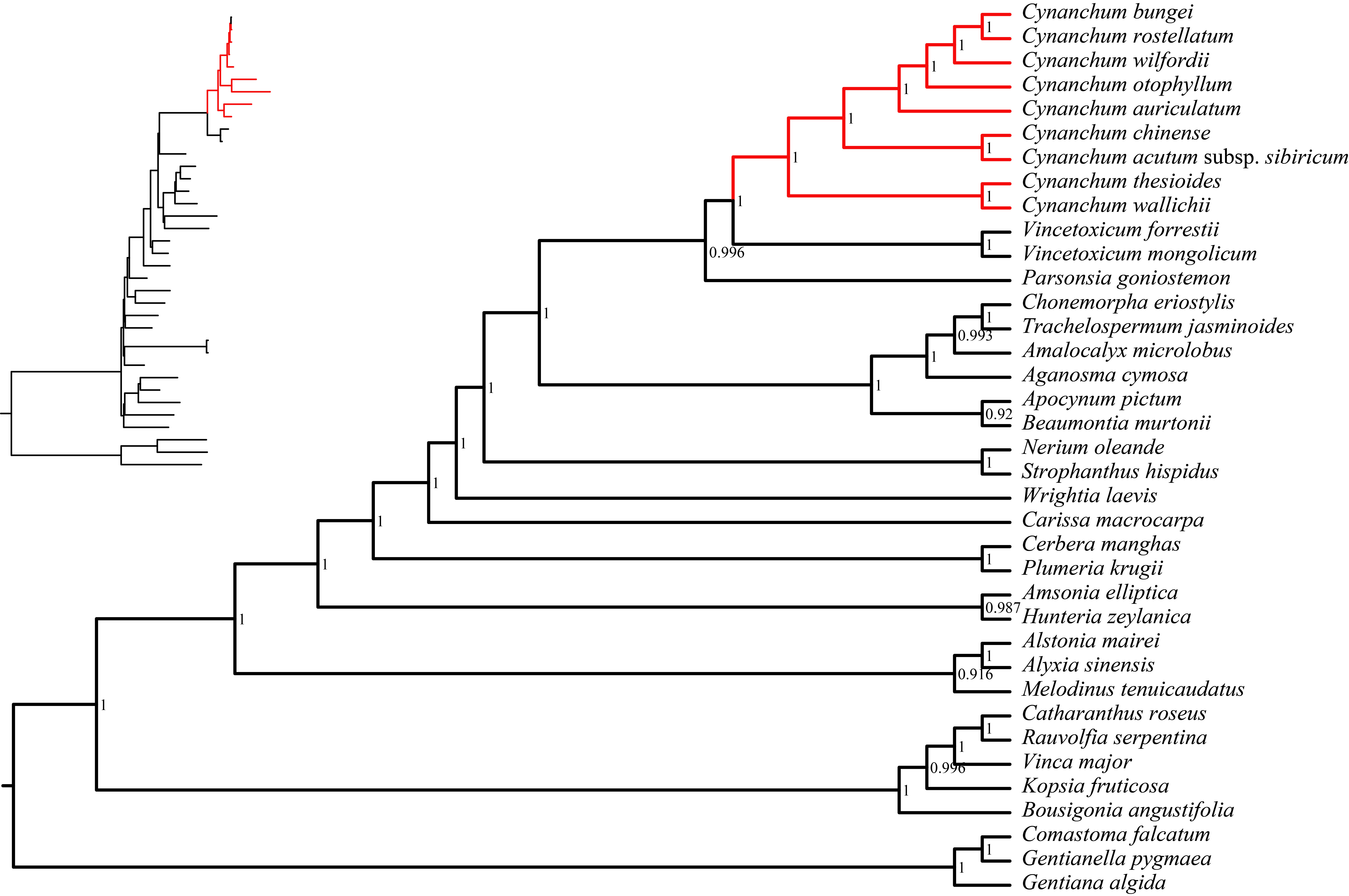

Supplement: Supplementary file 1 [file genes-15-00884-s001.zip › Figure S5.jpg]
